# Neurotransmitter content heterogeneity within an interneuron class shapes inhibitory transmission at a central synapse

**DOI:** 10.3389/fncel.2022.1060189

**Published:** 2023-01-04

**Authors:** Dimitri Dumontier, Caroline Mailhes-Hamon, Stéphane Supplisson, Stéphane Dieudonné

**Affiliations:** Institut de Biologie de l’Ecole Normale Supérieure (IBENS), École Normale Supérieure, Université PSL, CNRS, INSERM, Paris, France

**Keywords:** cerebellum, co-transmission, golgi cells, granule cells, inhibition

## Abstract

Neurotransmitter content is deemed the most basic defining criterion for neuronal classes, contrasting with the intercellular heterogeneity of many other molecular and functional features. Here we show, in the adult mouse brain, that neurotransmitter content variegation within a neuronal class is a component of its functional heterogeneity. Golgi cells (GoCs), the well-defined class of cerebellar interneurons inhibiting granule cells (GrCs), contain cytosolic glycine, accumulated by the neuronal transporter GlyT2, and GABA in various proportions. By performing acute manipulations of cytosolic GABA and glycine supply, we find that competition of glycine with GABA reduces the charge of IPSC evoked in GrCs and, more specifically, the amplitude of a slow component of the IPSC decay. We then pair GrCs recordings with optogenetic stimulations of single GoCs, which preserve the intracellular transmitter mixed content. We show that the strength and decay kinetics of GrCs IPSCs, which are entirely mediated by GABA_A_ receptors, are negatively correlated to the presynaptic expression of GlyT2 by GoCs. We isolate a slow spillover component of GrCs inhibition that is also affected by the expression of GlyT2, leading to a 56% decrease in relative charge. Our results support the hypothesis that presynaptic loading of glycine negatively impacts the GABAergic transmission in mixed interneurons, most likely through a competition for vesicular filling. We discuss how the heterogeneity of neurotransmitter supply within mixed interneurons like the GoC class may provide a presynaptic mechanism to tune the gain of microcircuits such as the granular layer, thereby expanding the realm of their possible dynamic behaviors.

## Introduction

Cellular neuroscience was founded on the morphological identification of neuronal classes, based on their dendritic and axonal morphologies, as revealed by Golgi staining. Refining this classification using electrophysiological and molecular profiles and assigning functions to individual neuron classes is still a central effort of modern neuroscience. However, adding more parameters to the description of neurons deceptively resulted in conflicting class separations (Petilla Interneuron Nomenclature et al., 2008; Tremblay et al., 2016; Zeng and Sanes, 2017), leading in the most extreme cases to a fractal view of neuronal populations (Parra et al., 1998; Hobert et al., 2016). Recently, single-cell RNA sequencing approaches have enabled clustering into broad neuronal classes, within which the large residual intra-class variability may not be easily reconciled with previously identified subpopulations (Zeisel et al., 2015; Tasic et al., 2016; Saunders et al., 2018; Kozareva et al., 2021).

Amidst this complexity, the neurotransmitter released by each neuron has been used early and is still considered a reliable criterion for defining broad neuronal classes. It delineates excitatory, inhibitory, and neuromodulatory neuronal populations, and has been extended to neuropeptides, such as somatostatin and VIP, to classify forebrain interneurons (Kepecs and Fishell, 2014; Tremblay et al., 2016; Lim et al., 2018). However, the prevalence of neurons that release multiple neurotransmitters or neuromodulators (Granger et al., 2017) has somewhat blurred this simple view, opening the possibility for a morpho-functional neuronal class to contain cells with quantitatively or qualitatively different mixtures of neurotransmitters. This constitutes a dazzling prospect, whereby the synaptic output of each presynaptic neuron within a class, defined by its neurotransmitter mixture, could be coordinated with its other properties such as its excitability or sensitivity to neuromodulation.

Varied cytosolic accumulation and synaptic co-release of GABA and glycine, as occurs in the majority of hindbrain inhibitory neurons, constitutes the most studied case of variability in neurotransmitter content (Jonas et al., 1998; Chery and de Koninck, 1999; O’Brien and Berger, 1999; Dumoulin et al., 2001; Awatramani et al., 2005; Dugue et al., 2005; Giber et al., 2015; Moore and Trussell, 2017; Nerlich et al., 2017). GABAergic, glycinergic and mixed inhibitory neurons persist into adulthood, when they can contact the same postsynaptic neurons (Ottersen et al., 1988; Todd and Sullivan, 1990; Dumba et al., 1998; Riquelme et al., 2001; Lu et al., 2008; Batten et al., 2010; Dufour et al., 2010; Husson et al., 2014; Paik et al., 2019). However, to date, the diversity of IPSCs properties has always been attributed to the expression by the postsynaptic neurons of different types of GABA_A_ and glycine receptors (Rossi and Hamann, 1998; Chery and de Koninck, 1999; Chery and De Koninck, 2000; Mitchell and Silver, 2000; Dumoulin et al., 2001; Gao et al., 2001; Keller et al., 2001; Ahmadi et al., 2003; Awatramani et al., 2005; Gonzalez-Forero and Alvarez, 2005; Lu et al., 2008; Rousseau et al., 2012; Giber et al., 2015; Moore and Trussell, 2017; Nerlich et al., 2017). While presynaptic and postsynaptic co-maturation has been reported (Nabekura et al., 2004), the role of presynaptic transmitter content in the properties and strength of mature inhibitory synapses has been overlooked. It remains to be formally investigated whether inhibitory neurons of the same morpho-functional class use different GABA/glycine mixture and how this mixture alters their postsynaptic action.

To address this question, we studied a well-defined morphological class of interneurons in the cerebellar cortex, the Golgi cells (GoCs), which exhibit stereotyped morphology and connectivity (Dieudonne, 1998; Galliano et al., 2010) but a diversity in neurotransmitter content (Ottersen et al., 1987, 1988; Dugue et al., 2005). GoCs cover the whole spectrum of inhibitory phenotypes, with a fifth of pure GABAergic neurons expressing only the GABA synthesis enzyme (glutamate decarboxylase GAD), a majority of mixed GoCs expressing in addition the neuronal glycine transporter, GlyT2, and a handful of pure glycinergic cells expressing GlyT2 but not GAD (Ottersen et al., 1987; Simat et al., 2007). This diversity is also observed at axonal varicosities of GoCs in cerebellar glomeruli (Ottersen et al., 1988; Dugue et al., 2005), where they synapse on billions of granule cells (GrCs) and on rare unipolar brush cells (UBCs). Studies of inhibition between GoCs and UBCs (Dugue et al., 2005; Rousseau et al., 2012) have demonstrated a major role for differential expression of GABA_A_Rs and GlyRs by postsynaptic UBCs in shifting transmission from a mixed system to a pure GABAergic system, with no evidence of an impact of presynaptic neurochemistry (Rousseau et al., 2012), as in other mixed systems to date. Remarkably, GrCs express only GABA_A_R, with two well-established subunit compositions: low-affinity synaptic receptors mediating fast IPSCs and high-affinity extrasynaptic receptors mediating a slow spillover component (Kaneda et al., 1995; Wall and Usowicz, 1997; Rossi and Hamann, 1998; Dumoulin et al., 2001). The absence of postsynaptic GlyR constitutes somewhat counterintuitively, by eliminating the dominant effect of postsynaptic receptor variability, an asset to explore the functional impact of presynaptic GABA/glycine content diversity.

Alteration and restoration of GlyT2 and GAD activities dynamically change the cytoplasmic content of glycine and GABA and their vesicular release, thus affecting postsynaptic action of inhibitory neurons (Rousseau et al., 2008; Apostolides and Trussell, 2013). However, the functional consequences of glycine accumulation in the subset of GoCs expressing GlyT2 remains unexplored at GrCs synapses because the GoC-GrC IPSCs are purely GABAergic. In this paper, we show that manipulating the relative supply of GABA and glycine to GoCs affects the charge content and decay kinetics of the compound GABA_A_ IPSCs evoked in granule cells by the electrical stimulation of the local GoCs. This provides evidence that the accumulation of glycine by presynaptic GoCs decreases the GABAergic inhibitory strength received by GrCs, most likely by competition of glycine with GABA for vesicular filling at GoCs terminals. To study if this mechanism is at work under normal transmitter supply conditions in GlyT2 expressing GoCs, we performed optogenetic stimulations of phenotypically identified GlyT2(+) or GlyT2(−) GoCs, paired with whole-cell GrCs recordings. We show that GlyT2 expression in GoCs is correlated with a reduced synaptic strength of the GrC GABA_A_ IPSCs at the level of their peak amplitude, decay kinetics and slow spillover charge. We thus show that variability in transmitter content within a neuronal class is an important component of the functional variability of that class and discuss the implications of this finding in the context of the granular layer computations.

## Results

### Extracellular glycine uptake by GlyT2 decreases GABAergic transmission to granule cells

**FIGURE 1:**
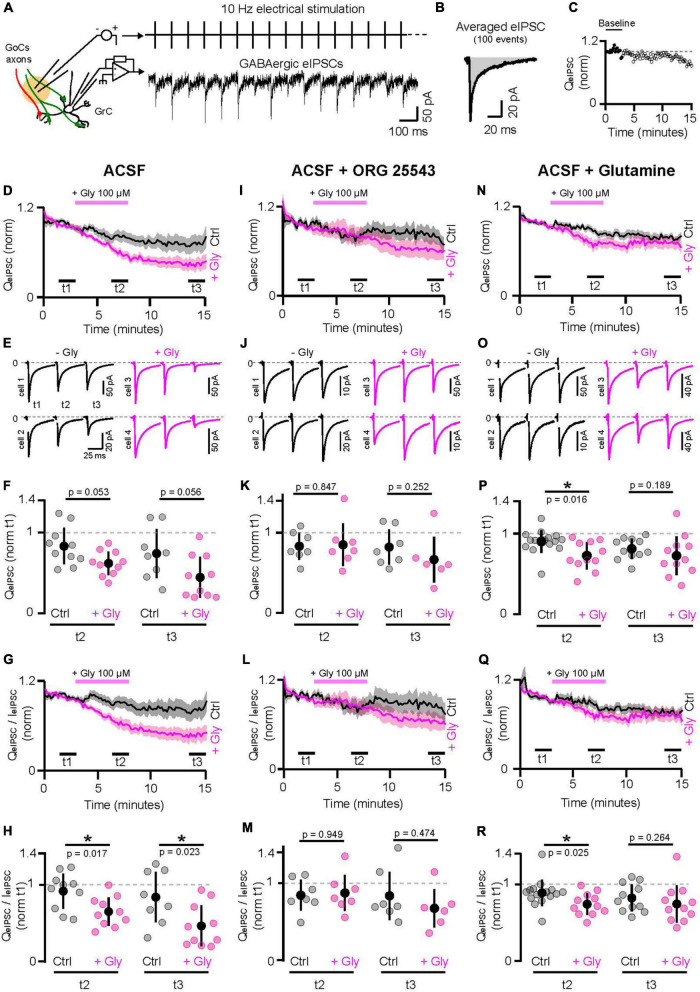
Manipulation of GABA and glycine supply to GoCs affect GrCs inhibition. **(A)** Left, schematic of the experiment. GABAergic IPSCs recorded from GrCs in whole cell configuration are evoked by the continue electrical stimulation *(eIPSC)* at 10 Hz of GlyT2(−) and GlyT2(+) GoCs axons without any distinction. Right, a GrC recording during 10 Hz stimulation of the GoCs axons. GABAergic eIPSCs have been pharmacologically isolated with the following cocktail of blockers: APV 50 μM, NBQX 2 μM, strychnine 0.5 μM, CGP55845 1 μM, and ORG24598 1 μM. **(B)** Average of 100 consecutive GrC eIPSCs following the electrical stimulation. The strength of the GABAergic transmission over the time is estimated by taking the charge (QeIPSC, gray area) of the averaged eIPSCs every 10 s. **(C)** Example of the QeIPSC over the time. Each dot was normalized by the mean signal in the baseline (3 min from the beginning, full dots). **(D)** Populational evolution of the GABAergic transmission over the time in ACSF (ctrl, black, *n* = 8–10) or ASCF + glycine 100 μM for 5 min (+Gly, pink, *n* = 10). Traces represent the mean normalized charge ± SEM. **(E)** Average of 1000 eIPSCs at t1, t2, and t3 in ctrl (black) and with glycine application *(pink)*. Two representative pairs are shown for each condition. **(F)** Charge measured from 1000 consecutive eIPSCs averaged at the end of the glycine application (t2) and at the end of the recordings (washout, t3), normalized by the baseline (t1). **(G,H)** Same as panels **(D,F)** using the charge to peak ratio *(QeIPSC/IeIPSC)*. **(I–M)** Same as panels **(D–H)** in presence of ORG25543 1 μM, the specific blocker of GlyT2 (ctrl *n* = 7, +Gly *n* = 6–7). **(N–R)** Same as panels **(D–M)** in ACSF supplemented with 500 μM of Glutamine (Gln), the precursor of the GABA synthesis (ctrl *n* = 11–15, +Gly *n* = 11). *p < 0.05.

We first applied glycine in the bath to examine whether and how its cytosolic accumulation in GoC expressing GlyT2 altered pure GABA signaling at GoC-GrC synapses (Supplementary Figure 1A). GABAergic IPSCs were evoked by electrical stimulation of GoCs axons at 10 Hz (Figure 1A), a frequency corresponding to their firing behavior in rodents *in vivo* (Vos et al., 1999; Simpson et al., 2005; Holtzman et al., 2006a) and *in vitro* (Kanichay and Silver, 2008; Tabuchi et al., 2019). The charge of the average of 100 consecutive eIPSCs was monitored over time (QeIPSC, Figure 1B) and normalized by the baseline value during the first 3 min (Figure 1C). Bath application of glycine (100 μM) caused a gradual decrease in GABAergic transmission, which did not appear to saturate even after 5 min of application (Figure 1D). Activation of inhibitory glycine receptors on GoCs (Dumoulin et al., 2001) may occur during bath application of glycine despite the continuous presence of 0.5 μM strychnine in the bath. This putative glycine-induced hyperpolarization could cause a decrease in the efficiency of extracellular electrical stimulation, which could partly explain the negative effect of glycine application on synaptic transmission. To counter this hypothesis, we verified that the success rate of juxta-threshold electrical stimulation of GoCs axons, assessed by cell-attached somatic-recordings of back-propagated APs, was not affected by glycine application in the presence of strychnine (control: 0.59 ± 0.06; glycine: 0.68 ± 0.1, *p* = 0.46, *n* = 6, two-tailed Wilcoxon test).

As transmission undergoes a slow rundown over time (see section Materials and methods and Supplementary Figure 1B), controls without bath application of glycine were randomly performed (Figure 1D). We compared the average eIPSC charge at three time intervals, before (t1) and at the end of the glycine application (t2), and after 7 min of washing (t3). While the charge ratio at t2/t1 reduced by 17% in control (0.83 ± 0.22 pC, *n* = 10), a larger reduction (38%) was observed during glycine application (0.62 ± 0.14 pC, *n* = 10; *p* = 0.053), without apparent recovery after washout (charge ratio at t3/t1 in control: 0.74 ± 0.3 pC, *n* = 8; glycine: 0.44 ± 0.25 pC, *n* = 10; *p* = 0.056) (Figures 1E, F). Although the large cell-to-cell variability decreases the statistical robustness, it could be noted 25% (t2) and 41% (t3) larger reductions of GABA transmission in glycine compared to the control condition. As this decrease in total charge could either be due to smaller amplitude or faster decay of the IPSC, we computed and compared the IPSC weighted decay time constant (Figure 1G), which was significantly smaller (by 29%) in glycine (t2 to t1 ratio in control: 0.91 ± 0.22, *n* = 10; glycine: 0.64 ± 0.18, *n* = 10, *p* = 0.017, Figure 1H). Furthermore, the depression of charge to peak-amplitude by glycine applied in the bath did not significantly reverse after 7 min of washout (t3 to t1 ratio in control: 0.83 ± 0.32, *n* = 8; in glycine: 0.46 ± 0.26, *n* = 10; *p* = 0.023; 44.6% reduction, Figure 1H).

Co-release of GABA can speed up glycinergic currents in mixed GABA-glycine synapses of the auditory brainstem by acting as a non-competitive antagonist (Lu et al., 2008), but the converse interaction of glycine on GABA_A_ receptors has not been demonstrated. To confirm that bath-applied glycine does not induce functional cross talk on the GABA_A_ receptors, we show that the decay time constants of the eIPSCs at t2, with or without glycine are not different (control Tau1: 3.48 ± 1.2 ms, Tau2: 36.5 ± 7.7 ms, *n* = 32; glycine Tau1: 3.19 ± 1.3 ms, Tau2: 36.7 ± 8.3 ms, *n* = 29; Tau1 control vs. glycine: *p* = 0.2, Tau2 control vs. glycine: *p* = 0.79; Supplementary Figures 1C–E). However, despite unmodified decay time constants, GABAergic IPSCs evoked in the presence of glycine had a decreased relative amplitude of their second decay component, as compared to control [ctrl A2/(A1 + A2), t1: 0.5 ± 0.11, t2: 0.47 ± 0.16, *p* = 0.496; glycine A2/(A1 + A2), t1: 0.49 ± 0.14, t2: 0.40 ± 0.12, *p* = 0.015; two-tailed Wilcoxon test; Supplementary Figure 1C]. In agreement with the charge and weighted time constant decreases, we found that the charge of the synaptic current integrated from 36 ms after the eIPSC peak, when the fast component has already fully decayed, was reduced in the presence of glycine (Slow charge in control, t1: 0.28 ± 0.12 pC, t2: 0.21 ± 0.08 pC, *p* = 0.307; Slow charge in glycine, t1: 0.29 ± 0.09 pC, T2: 0.17 ± 0.06 pC, *p* = 0.007).

To confirm that the negative effect of glycine on GABAergic transmission of GrCs involves GlyT2 uptake, we added its specific blocker, ORG25543 (1 μM), during pre-incubation of slices and continuously during the experiment. Under this condition, glycine application did not cause a reduction in charge (t2 to t1 ratio in control: 0.83 ± 0.17, *n* = 7; in glycine: 0.85 ± 0.26, *n* = 7, *p* = 0.847; t3 to t1 ratio in control: 0.82 ± 0.22, *n* = 7; in glycine: 0.66 ± 0.28, *n* = 6; *p* = 0.252; Figures 1I–K) nor in the charge-to peak ratio (t2 to t1 ratio in control: 0.84 ± 0.19, *n* = 7; in glycine: 0.87 ± 0.23, *n* = 7, *p* = 0.949; t3 to t1 ratio in control: 0.83 ± 0.31, *n* = 7; in glycine: 0.67 ± 0.24, *n* = 6; *p* = 0.474; Figures 1L, M). In these conditions, the relative amplitude of the second decay component and the charge of the slow component were not found to be significantly different in the presence of glycine (ctrl A2/A1 + A2, t1: 0.49 ± 0.11, t2: 0.50 ± 0.13, *p* = 0.84; glycine A2/A1 + A2, t1: 0.56 ± 0.10, t2: 0.52 ± 0.11, *p* = 0.065; two-tailed Wilcoxon test; Supplementary Figure 1D; Slow charge in control, t1: 0.14 ± 0.046 pC, t2: 0.10 ± 0.03 pC, *p* = 0.159; Slow charge in glycine, t1: 0.28 ± 0.19 pC, t2: 0.21 ± 0.14 pC, *p* = 0.44).

To further confirm that the effect of glycine was due to the presynaptic competition of glycine with GABA, most likely for vesicular loading by their common transporter VIAAT which is the only known site of competition (Aubrey et al., 2007; Apostolides and Trussell, 2013), we sought to increase *de novo* synthesis of GABA by supplying extracellular glutamine, which is transported intracellularly and acts as a precursor of cytoplasmic GABA (Ishibashi et al., 2013; Wang et al., 2013). In ACSF supplemented with 500 μM glutamine, the extracellular application of glycine caused a reduced decrease in charges compared to control (t2 to t1 ratio in control: 0.9 ± 0.14%, *n* = 15; glycine: 0.72 ± 0.17%, *n* = 11; *p* = 0.016; Figures 1N–P). In addition, the small effect of glycine reversed to non-significant levels after 7 min of washout in the continuous presence of glutamine (t3 to t1 ratio in control: 0.81 ± 0.12%, *n* = 11, glycine: 0.72 ± 0.24%, *n* = 11; *p* = 0.189; Figures 1N–P). The charge-to-peak ratios show the same trend with a significant decrease in transmission but 1.7-fold smaller than without glutamine (t2 to t1 ratio in control: 0.88 ± 0.17, *n* = 15; glycine: 0.73 ± 0.14, *n* = 11, *p* = 0.025, 16.7% of decrease, Figures 1Q, R) and a reversion to non-significant levels after 7 min of glycine washout (t3 to t1 ratio in control: 0.82 ± 0.17, *n* = 11, glycine: 0.74 ± 0.24, *n* = 11, *p* = 0.264; Figures 1Q, R). Accordingly, we found that the relative amplitude of the second decay component is not affected by glycine in these conditions (ctrl A2/(A1 + A2), t1: 0.47 ± 0.11, t2: 0.45 ± 0.13, *p* = 0.074; glycine A2/(A1 + A2), t1: 0.59 ± 0.16, t2: 0.59 ± 0.17, *p* = 0.7; two-tailed Wilcoxon test; Supplementary Figure 1E). Moreover, glycine application had a minor effect on the slow component strength in the presence of glutamine (slow charge in control, t1: 0.24 ± 0.15 pC, t2: 0.20 ± 0.14 pC, *p* = 0.48; slow charge in glycine, t1: 0.28 ± 0.10 pC, t2: 0.19 ± 0.08 pC, *p* = 0.048). Somehow, these results recapitulate the effects obtained on mixed IPSCs by alteration of cytoplasmic GABA/glycine content (Apostolides and Trussell, 2013), except that the GlyR-mediated component of IPSCs is absent here.

### A targeted optogenetic stimulation strategy for GoC-GrC paired recordings without presynaptic dialysis

**FIGURE 2:**
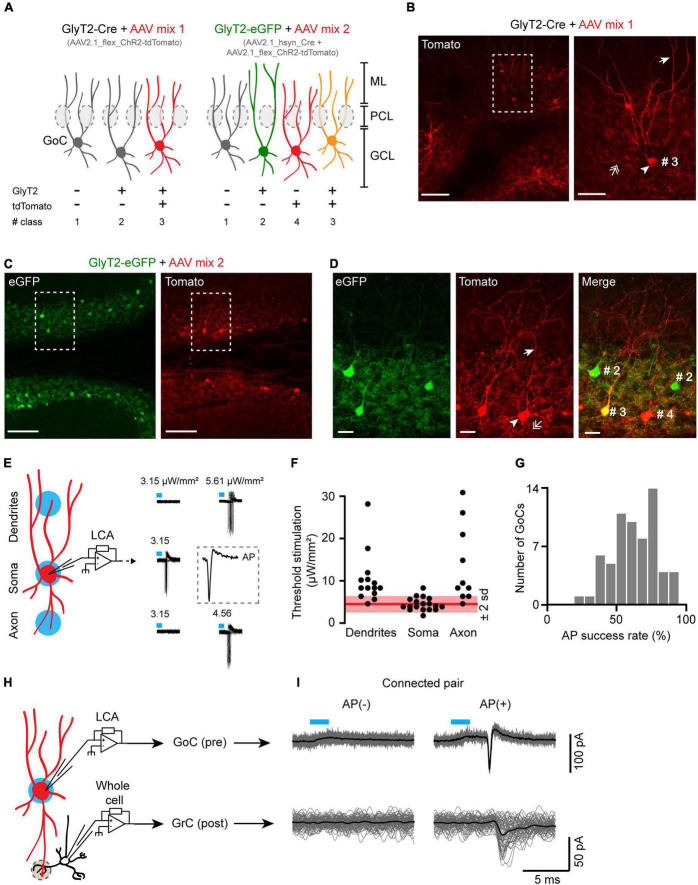
A targeted optogenetic stimulation strategy for GoC-GrC paired recordings without presynaptic dialysis. **(A)** Schematic of the viral strategies to express the channelrhodopsin (ChR2) fused with the tdTomato as reporter in GlyT2(+) and GlyT2(−) GoCs. GCL, granular layer; PCL, Purkinje cells layer; ML, molecular layer. **(B)** Slice of a representative GlyT2-Cre mouse injected with the mix 1 of AAV. Left panel, apex of the lobule IV showing infected, tdTomato(+) GoCs. Scale bar: 100 μm. Right panel left panel magnification showing the characteristic GoCs morphology. Arrowhead: soma; arrow: apical dendrite; double arrow: axons. Scale bar: 20 μm. **(C)** Same as panel **(B)** for a GlyT2-eGFP mouse injected with the mix 2 of AAV. Scale bar: 100 μm. **(D)** Magnification of panel **(C)**. (#2 and #3): GlyT2(+) GoC; (#4): GlyT2(−) GoC. Scale bar: 20 μm. **(E)** Left panel, calibration of the optogenetic stimulation power (in blue) to reach >80% of AP success (Threshold stimulation). Right panel, threshold stimulation power at the soma, dendrites, and axons of a representative GlyT2-Cre(+) GoC. Insert, magnification of an AP evoked at the soma. **(F)** Populational distribution of the threshold stimulation power (dendrites *n* = 13, soma *n* = 18, axons *n* = 10). In red, mean power at the soma ±2 SD. **(G)** Histogram of AP success rate evoked by somatic subthreshold stimulation in connected and unconnected GlyT2(+) and GlyT2(−) GoCs (*n* = 64). **(H)** Schematic of the GoC-GrC optogenetic pairs recording of the GABAergic transmission in cerebellar glomeruli (gray dotted circle). **(I)** Representative example of a specifically connected GoC-GrC pair.

Although our results argue for a global negative effect of GlyT2 expression and subsequent glycine accumulation onto GoCs-GrCs communication in the adult cerebellum, the use of electrical stimulation does not allow to distinguish the relative contributions of GlyT2(+) and GlyT2(−) GoCs. We then investigated whether the build-in of glycine by GlyT2 under physiological conditions, i.e., when extrinsic glycine is not added, affects the strength of synaptic inhibition received by GrC. To this end we sought to record pairs between neurochemically identified GoCs and their synaptically connected GrC targets, in order to compare the IPSCs generated by GoCs expressing GlyT2 to those generated by the GlyT2(−) GoCs, considered as a negative control. Whole-cell pair recordings, which represent the technique of choice to characterize synaptic connections, cannot be used in this case, because dialysis of the cytoplasm of the presynaptic GoCs would alter their neurotransmitter content and synaptic physiology (Diana and Marty, 2003; Wang et al., 2013). To address this issue, we implemented targeted optogenetic stimulations of GoCs. Channelrhodopsin (ChR2) expression in identified GoCs was achieved by stereotactic injections of CRE-dependent AAV2.1 virus into the cerebellum of GlyT2-Cre or GlyT2-eGFP mice (Figure 2A). Injection of AAV2.1_CAGGS_Flex_ChR2_td-Tomato_WPRE_SV40 into GlyT2-Cre transgenic mice targeted GlyT2(+) cells (Mix1, Figure 2B). Co-injection of AAV2.1-HSyn-Cre_WPRE_hGH with AAV2.1_CAGGS_Flex_ChR2_td-Tomato_WPRE_SV40 in GlyT2-eGFP mice enabled expression in all GoC subtypes (Mix2, Figure 2C). In this second strategy, GlyT2(−) GoCs are identified by the absence of eGFP expression (Figure 2D), which has been previously confirmed to coincide with an absence of GlyT2 transport current (Rousseau et al., 2008). On the other hand, GlyT2(+) GoCs are identified by the eGFP signal that is a reporter of the glycinergic phenotype of neurons in this line (Dugue et al., 2005; Zeilhofer et al., 2005; Rousseau et al., 2008). GoCs were easily recognized by their large somas in the granular layer and characteristic apical dendrites in the molecular layer (Figures 2B, D). GlyT2(+) optogenetic pairs were identified and recorded in adult cerebellar slices expressing the AAV Mix1 in GlyT2-CRE, while GlyT2(−) optogenetic pairs were only identified using AAV Mix2 in GlyT2-eGFP cerebellar slices.

Optogenetic stimulations of GoCs using a restricted illumination field (20–30 μm in diameter) on the cell soma have previously been performed (Crowley et al., 2009). However, the specificity of optogenetic stimulations needs to be validated, as dendrites and axons of other GoCs (ChR2+) could cross the stimulation light beam above or below the targeted soma, as illustrated by the extensive neuritic expression of td-Tomato(+) in the granular layer in our GoC expression paradigm (Figures 2B, C). We therefore calibrate the optogenetic stimulation on slices of GlyT2-Cre animals expressing ChR2 in GlyT2(+) GoCs by viral transgenesis. We recorded GoCs in the cell-attached configuration and defined a stimulation threshold for each targeted cell as the lowest light intensity to evoke >80% of AP (Figure 2E). This stimulation threshold was always lower when the illumination spot was centered on the soma (4.47 ± 1.5 μW/mm^2^, *n* = 18) than when placed remotely on the dendritic (10.3 ± 6 μW/mm^2^, *n* = 13) or axonal fields (13.6 ± 8.8 μW/mm^2^, *n* = 10) of the same GoC (soma vs. dendrites: *p* < 0.001, soma vs. axon: *p* < 0.001, Wilcoxon’s bilateral test). However, the liminal power for some of the less excitable somas of the population could be strong enough to trigger a spike by illumination of the axons or dendrites of the most excitable GoCs (Figure 2F). Thus, synaptic connections evoked by optical stimulation at the threshold intensity could well come from stimulation of axons and dendrites of nearby GoCs rather than from the soma of the targeted GoC.

As there is *a priori* no guarantee that IPSCs evoked by efficient somatic stimulation originate exclusively from the target cell, we reduced and set the intensity of the optogenetic stimulation around the AP threshold (Spike success rate: 62 ± 16%, *n* = 64; Figure 2G). To systematically verify the specificity of this subthreshold optogenetic stimulation during paired recordings of GoCs and GrCs, we recorded light-evoked action potentials from the targeted GoC with a loose cell-attached patch pipette placed on its soma (Figure 2H), and we checked that AP failure in the GoC always resulted in IPSC failure in the postsynaptic GrC (Figure 2I).

### GlyT2(−) and GlyT2(+) GoC-GrC connections have different synaptic properties

**FIGURE 3:**
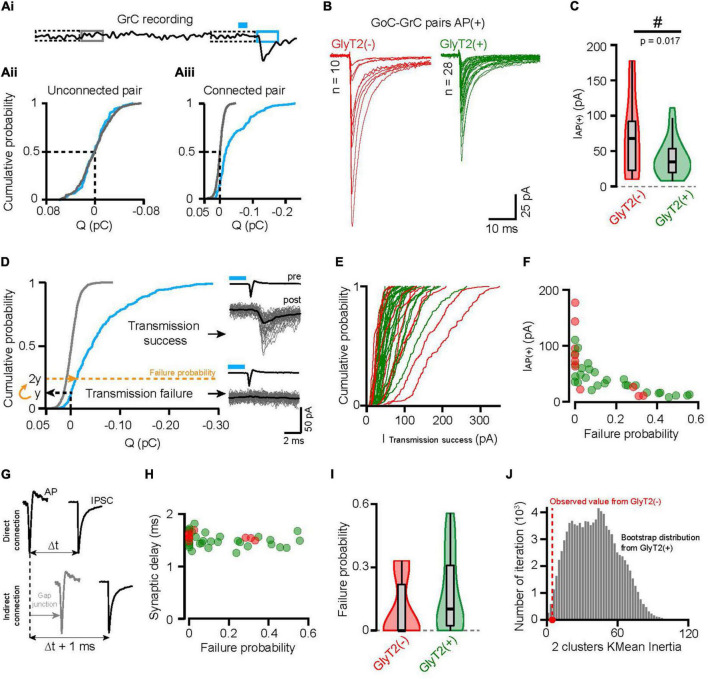
GlyT2(–) and GlyT2(+) GoC-GrC connections have different synaptic properties. **(Ai)** Illustration of the charge measurement in GrCs recordings before (gray) and after optogenetic stimulation evoking GoCs AP *(blue)*. Dotted rectangles represent the local 10 ms normalization windows (see materials and method). **(Aii)** Cumulative probability distribution of the baseline (gray) and post-AP (blue) charges from a representative unconnected pair. **(Aiii)** Same as panel **(Aii)** for a representative connected pair. **(B)** Average GrCs traces following optogenetically evoked AP in GyT2(–) (red, *n* = 10) and GlyT2(+) (green, *n* = 28) GoCs. These traces will be referred as AP(+) from here and I_AP(+)_ is the peak amplitude of the averaged AP(+) traces. **(C)** Violin plot distribution of I_AP(+)_ of GlyT2(–) and GlyT2(+) pairs shown in panel **(B)**. The “#” symbolized the significative result from the Conover test of variance. **(D)** Same representation as panels **(Aii, Aiii)**, showing the transmission failure threshold of a representative connected pair (dotted yellow line). Right insert, resulting classification of the example traces in transmission success or failure with the corresponding average *(black)*. **(E)** Cumulative distribution probability of the transmission success peak amplitude for each GoC-GrC pairs. These curves show that GlyT2(–) pairs are more right shifted than the GlyT2(+) ones, but no significant differences emerge with this metric cleaned of transmission failure [GlyT2(–): 80.78 ± 51.75 pA, *n* = 10; GlyT2(+): 53.94 ± 23.75 pA, *n* = 28; *p* = 0.3]. **(F)** Transmission failure probability in function of the I_AP(+)_ for all connected pairs (*n* = 38). **(G)** Schematic illustrating the consequence of indirect GoC-GrC connection through Gap junction on the synaptic delay. **(H)** Transmission failure probability in function of the synaptic delay (peak-to-peak time between AP and I_AP(+)_; GlyT2(–): 1.57 ± 0.072 ms, *n* = 10; GlyT2(+): 1.49 ± 0.133 ms, *n* = 28; *p* = 0.04). **(I)** Violin plot distribution of failure probability showing a bimodal distribution for GlyT2(–) GoCs pairs and a continue one for GlyT2(+) ones. **(J)** Histogram of the inertia for 2 clusters *K*-mean analysis on bootstrap distribution of GlyT2(+) failure probability distribution *(green)*. The red dot is the inertia of GlyT2(–) 2 clusters *K*-mean analysis on the distribution shown in panel **(I)**. This figure shows how likely 8 points draw randomly with replace from the GlyT2(+) distribution give rise to a distribution as separated as the GlyT2(–) one.

To quantify the synaptic currents in GrC whole-cell recordings with maximum sensitivity, we calculated the 5-ms time integral of the current starting 1 ms after the GoC spike, and after zeroing the average current in the preceding 10 ms (Figure 3Ai). As expected, the integrated charge sampled randomly from the baseline before stimulation followed a very narrow distribution centered on zero (Figures 3Aii, Aiii, gray solid line, see technical details in section Materials and methods). In 26 of 64 pairs, the post-AP distribution of charge was not significantly different from the baseline distribution (Figure 3Aii), indicating unconnected pairs (Kolmogorov-Smirnov test *p* > 0.05), whereas the distribution was highly significantly right-shifted in the remaining 38 connected pairs (Figure 3Aiii, Kolmogorov-Smirnov test, *p* < 0.0001).

The amplitude of the mean IPSC, evoked by effective optogenetic stimulation that triggered an action potential in the presynaptic GoCs, was highly variable between connected pairs (48.4 ± 37.8 pA, *n* = 38 pairs, CV = 0.78; Figures 3B, C), and shows distinct distributions for GlyT2(−) GoCs (70.3 ± 53.9 pA, *n* = 10) and GlyT2(+) GoCs (40.6 ± 26 pA, *n* = 28; Conover test of equal variance: *p* = 0.017; Figure 3C). The GlyT2(−) and GlyT2(+) amplitude distributions are approximately proportional both in mean and variance with ratios of 1.73 and 2.07, respectively. This is consistent with a larger vesicular transmitter content at GlyT2(−) GoC synapses. However, the mean amplitude of GlyT2(+) GoC IPSCs was not found statistically significantly lower than that of GlyT2(−) GoC IPSCs (*p* = 0.28), which is to be expected, given the large CVs and skewness of the distributions.

### Different connectivity rules govern GlyT2(−) and GlyT2(+) GoCs synaptic contacts with GrCs

The transmission failure rate is an important quantal parameter of synaptic transmission, indicative of the number of active sites and the probability of vesicular release. The large difference between the synaptic and baseline charge distribution in connected pairs separated transmission successes from failures (Figure 3D). We set the postsynaptic failure probability for synaptic currents to twice the probability of measuring positive charge (outward current) (Figure 3D), knowing that the charge distribution of failures is symmetric around zero in the baseline (Figures 3Aii, Aiii). This limit defines a conservative estimate of the failure rate (0.16 ± 0.18, *n* = 38) since all traces below this threshold show no IPSC waveform (Figure 3D). Eliminating transmission failure does not change the broad distribution of IPSC amplitude for each connected pair (*p* = 0.3), suggesting the contribution of multiple sources of variability (Figure 3E).

The occurrence of pairs with a high probability of transmission failure and a low amplitude IPSC (10 pairs out of 38 have a transmission failure probability between 0.3 and 0.55 in both GlyT2(−) and GlyT2(+) GoCs populations; Figure 3F) drew our attention because of a possible artifact that could confound our analysis. Indeed, it is well established that a fraction of the AP evoked in a GoC can propagate to neighboring GoCs through electrical gap junctions, emulating an indirect connection with low release probability (Figure 3G; Dugue et al., 2009; Vervaeke et al., 2010). To rule out this possibility, we measured the latency of evoked IPSCs with respect to the peak of the GoC AP, knowing that spike propagation *via* gap junctions introduces an additional delay of ∼1 ms (Dugue et al., 2009; Vervaeke et al., 2010). In our data, the mean synaptic delay had a clear monomodal distribution (1.51 ± 0.125 ms, *n* = 38), and did not correlate with the probability of transmission failure (*r* = −0.25, *p* = 0.11, *n* = 38, Figure 3H), confirming the pre-post synaptic specificity of our recorded pairs and the validity of our failure probability measurements.

Failure rates were on average lower for GlyT2(−) pairs but not significantly different from GlyT2(+) ones (GlyT2(−): 0.095 ± 0.14, *n* = 10; GlyT2(+): 0.18 ± 0.18, *n* = 28; *p* = 0.061; Figure 3I). However, *K*-means analysis shows that the failure rate of GlyT2(−) GoCs follows a bimodal distribution whereas this does not seem to be the case for GlyT2(+) GoCs (*k* = 2 clusters explain 99 and 80.5% of their dispersion, respectively). Bootstrap analysis performed by drawing 10 samples from the GlyT2(+) distribution and calculating their K-means inertia shows that the probability that the inertia of the two clusters from the GlyT2(−) distribution is explained by a different drawing of the GlyT2(+) distribution is 0.8% (one-sided) (Figure 3J; see details in the section Materials and methods). Together, these results (Figures 3B–F) show that GlyT2(−) GoCs follow different synaptic connectivity rules than GlyT2(+) GoCs.

### Different types of IPSCs reveal decreased and variable GABA transients at GlyT2(+) synapses

In Figure 1, the main effect of loading GlyT2(+) GoCs with glycine through bath application was to decrease the proportion of the slow component of the IPSCs decay, thus reducing the charge to amplitude ratio. We thus examined and compared the charges and kinetics of IPSCs evoked by optogenetic stimulations of GlyT2(+) and GlyT2(−) GoCs.

**FIGURE 4:**
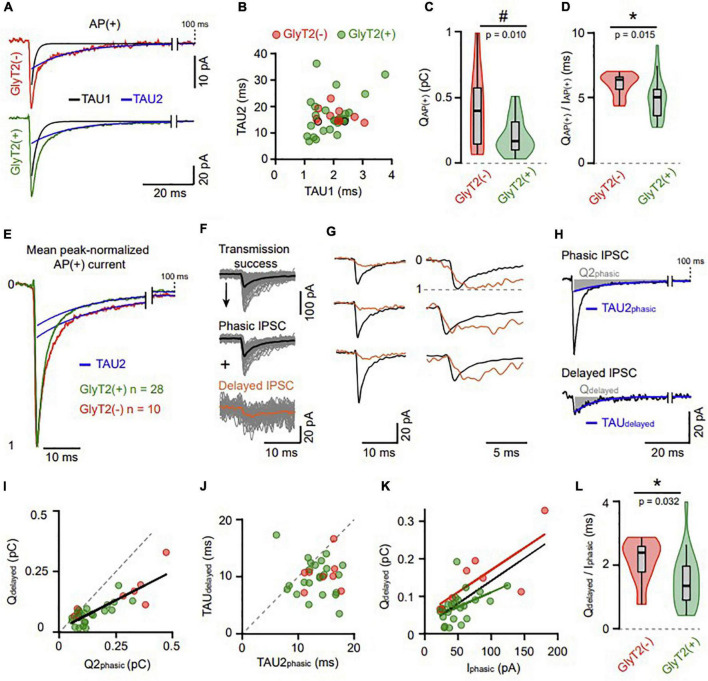
Different types of IPSCs unravel decreased and variable GABA transient at GlyT2(+) synapses. **(A)** Example of GlyT2(−) and GlyT2(+) AP(+) averaged traces both adjusted by a bi-exponential function. The two decay components have a fast *(black)* and a slow *(blue)* time constant named TAU1 and TAU2, respectively. **(B)** Correlation between the two fitted time constants in connected GoC-GrC pairs [*n* = 36, GlyT2(−) *n* = 10, GlyT2(+) *n* = 26]. The two dots outlined in black are the individual examples in panel **(A)**. GlyT2(−): TAU1 = 2.04 ± 0.51 ms, TAU2 = 16.56 ± 2.73 ms, *n* = 10; GlyT2(+): TAU1 = 1.94 ± 0.62 ms, TAU2 = 16.41 ± 7.17 ms, *n* = 26; TAU1 GlyT2(−) vs. TAU1 GlyT2(+), *p* = 0.46, TAU2 GlyT2(−) vs. TAU2 GlyT2(+), *p* = 0.58. **(C)** Violin plot distribution of the synaptic charge [Q_AP(+)_] calculated from bi-exponential fit (A1*TAU1 + A2*TAU2) of mean AP(+) current. The “#” indicates a significant difference for the Conover test of variance. **(D)** Violin plot distribution of the ratio between QAP(+) and the peak amplitude of AP(+) current [I_AP(+)_] (Figure 2B). **(E)** Mean peak-normalized AP(+) current of the GlyT2(−) and GlyT2(+) pairs (*n* = 10 and *n* = 28, respectively). The superposed blue traces are the second decay component from the bi-exponential function [same fitting procedure as panel **(A)**]. **(F)** Transmission success from a representative pair decomposed in phasic (average trace in black) and delayed (average trace in orange) events (see details in section Materials and methods). **(G)** Examples exhibiting kinetics difference of phasic and delayed events (left: average traces; right: peak-normalized traces). **(H)** Average phasic and delayed IPSC of a pair adjusted with a bi- and a mono-exponential function reciprocally. In blue, the second decay component of the phasic IPSC (TAU2_phasic_) and the single decay component of the delayed IPSC *(TAU_delayed_)*. The areas shaded in gray represent the charge of the corresponding decay component in phasic (Q2_phasic_ = A2*TAU2_phasic_) and delayed (Q_delayed_ = A*TAU_delayed_) events. The peak amplitude of the phasic IPSC *(I_phasic_)* did not differ significantly between GlyT2(+) and GlyT2(−) pairs. **(I)** Correlation between Q2_phasic_ and Q_delayed_. **(J)** Correlation between the decay time constant of delayed IPSCs (TAU_delayed_) and the second one of phasic IPSCs (TAU2_phasic_). **(I,J)** The gray dotted lines are diagonals, and exhibit a lack of charges and time in delayed IPSCs compared to the phasic ones. **(K)** Correlation between I_phasic_ and Q_delayed_ in the GlyT2(−) and GlyT2(+) population. In black the correlation of all pairs pooled together (slope = 1.2 ms, *r* = 0.66, *p* < 0.0001, *n* = 31). **(L)** Violin plot distribution of Q_delayed_ to I_phasic_ ratio for GlyT2(+) and GlyT2(−) pairs. These ratios are the slopes of each dot in panel **(K)** and depict that GlyT2(+) variability exceeds the one of GlyT2(−) pairs. *p < 0.05.

The decay of AP(+) IPSCs (including postsynaptic failures but excluding presynaptic stimulation failures) was well fitted by a bi-exponential function (unitary examples of each group are shown in Figure 4A), which yielded time constants characteristic of mature GoC-GC synapses (Tau1: 1.97 ± 0.6 ms, Tau2: 16.45 ± 6.26 ms, *n* = 36; Figure 4B; Brickley et al., 1996; Rossi and Hamann, 1998; Crowley et al., 2009). The two-time constants were not correlated (Tau1 vs. Tau2, *r* = 0.263, *p* = 0.12, *n* = 36; Figure 4B), and were almost identical between GoC types. However, the mean AP(+) charge was twofold higher for GlyT2(−) than GlyT2(+) GoCs [0.4 ± 0.3 pC (*n* = 10) and 0.2 ± 0.13 pC (*n* = 26), respectively; *p* = 0.1; Figure 4C], and their variances were not homogenous (Conover test, *p* = 0.010), suggesting again two scaled distributions, as previously found for I_AP(+)_ (Figure 3C). In agreement, the variance heterogeneity of the charge almost vanished after normalization by I_AP(+)_ (Figure 4D, Conover test of equal variance, *p* = 0.11). This Q_AP(+)_/I_AP(+)_ ratio establishes a mean Tau weighted that is 21% higher in the GoC GlyT2(−) group (GlyT2(−): 6.0 ± 0.82 ms, *n* = 10; GlyT2(+): 4.89 ± 1.5 ms, *n* = 26; *p* = 0.0154; Figure 4D) indicating prolonged activation of GABA_A_ receptors at this synapse. The mean trace of normalized IPSCs confirms a larger contribution of the second, slower, component of decay in the GlyT2(−) than in the GlyT2(+) population (A2/(A1 + A2): 0.281 vs. 0.219, respectively, Figure 4E), in agreement with the results of Glycine loading by bath application.

Close examination of individual traces (see section Materials and methods) revealed a subpopulation of low-amplitude, delayed IPSCs lacking the fast-rising fast-decaying component characteristic of phasic IPSCs (Figures 4F–H). These delayed IPSCs have similar prevalence in the two GoC populations [GlyT2(−): 23.5 ± 25.5%, *n* = 10; GlyT2(+): 26.2 ± 13.8%, *n* = 28, *p* = 0.28] and their decay was well fitted by a single exponential function (TAU_delayed_: 10 ± 3 ms, *n* = 31). The charge of the delayed IPSCs was twofold higher in GlyT2(−) pairs than GlyT2(+) ones [Q_delayed_ GlyT2(−): 0.147 ± 0.08 pC, *n* = 8; GlyT2(+): 0.073 ± 0.04 pC, *n* = 23; *p* = 0.0176, Figure 4I] and was highly correlated with the Q2_phasic_ component for each pair (slope = 0.48, *r* = 0.716, *p* < 0.0001, *n* = 31, Figure 4I). This reduced Q_delayed_, as compared with Q2_phasic_, was partly accounted for by a faster decay time constant (TAU_delayed_ vs. TAU2_phasic_, *p* < 0.0001, *n* = 31, two-tailed Wilcoxon test, Figure 4J). This would be consistent with a partial activation of the same clusters of synaptic receptors that mediate phasic IPSCs, because of a reduced and/or altered neurotransmitter transient (partly filled vesicle, distant release site, slow fusion…). Accordingly, Q_delayed_ correlates with I_phasic_ in the GlyT2(−) and GlyT2(+) groups, supporting a common postsynaptic receptor origin of the two types of synaptic currents [GlyT2(−): slope = 1.18 ms, *r* = 0.76, *p* = 0.028 *n* = 8; GlyT2(+): slope = 0.78 ms, *r* = 0.56, *p* = 0.005, *n* = 23; Figure 4K]. Thus, the Q_delayed_/I_phasic_ ratio was used as another postsynaptic estimator of the GABA transient evoking IPSCs and was again found to be higher in GlyT2(−) than GlyT2(+) GoCs (GlyT2(−): 2.1 ± 0.64 ms, *n* = 10; GlyT2(+): 1.5 ± 0.82 ms, *n* = 23; *p* = 0.032, Figure 4L).

### A stronger activation of high-affinity GABA_A_R by GlyT2(−) GoCs

**FIGURE 5:**
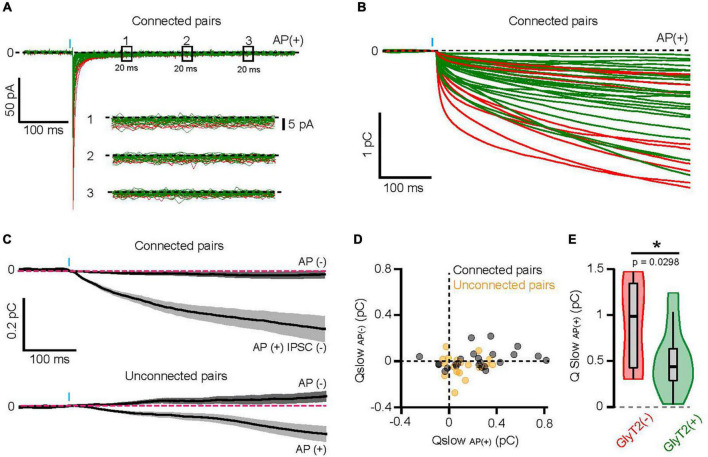
A stronger activation of high-affinity GABA_A_R by GlyT2(−) GoCs. **(A)** Full scale averaged IPSCs from GlyT2(−) and GlyT2(+) GoC-GrC paired recordings. Windows (1), (2), and (3) correspond to 20 ms of recording at 100, 200, and 300 ms from the IPSC peak, respectively. Insert shows the magnification of windows (1), (2), and (3) where the black dotted line represents 0 pA. **(B)** Cumulative sum *(integral)* of the mean AP(+) current for each GoC-GrC pairs (red = GlyT2(−), *n* = 10; green = GlyT2(+), *n* = 27). **(C)** Average integrals ± SEM for AP failure [AP(−)] or IPSC failure [AP(+) IPSC(−)] in connected (top, *n* = 24) and unconnected pairs (down, *n* = 18). **(D)** Slow component charges *(Qslow)* in AP(−) and AP(+) IPSC(−) mean current from connected (black, *n* = 24) and unconnected pairs (yellow, *n* = 18). **(E)** Violon plot distribution of Qslow _AP(+)_ in GlyT2(+) (*n* = 25) and GlyT2(−) (*n* = 10) pairs. *p < 0.05.

In addition to the fast IPSC, inhibitory transmission between GoCs and GrCs involves a slower component that is mediated by high-affinity extrasynaptic GABA_A_Rs activated by spillover and accumulation of GABA in cerebellar glomeruli (Rossi and Hamann, 1998; Mapelli et al., 2014). This volume transmission should be ideally suited to reveal the concentration of GABA released by Golgi cells in the glomeruli. In connected pairs, the time-integral of the average AP(+) traces (cumulated charge) revealed this slow component that prolongs the fast IPSC with a tail current of very low amplitude (Figures 5A, B), but which carries 2.5 ± 1.5 times more charges (0.62 ± 0.38 pC, *n* = 32, *p* < 0.0001, two-tailed Wilcoxon test) than the fast IPSC. We used long recording windows of 2 s in a subset of GoC-GrC pairs to fit the slow charge component (Q_slow_) with a mono-exponential function (tau_slow_ = 359 ± 111 ms, *n* = 6), within the range of kinetics of the high-affinity extrasynaptic GABA_A_R components recorded previously (Rossi and Hamann, 1998). As expected, Q_slow_ measured in traces without presynaptic AP did not differ from zero (0.009 ± 0.087 pC, *n* = 42), further confirming the specificity of our optogenetic pairs.

In connected pairs, Q_slow_ measured in the IPSC failure traces [AP(+) IPSC(−): 0.23 ± 0.25 pC] was smaller than in AP(+) IPSC(+) traces (0.6 ± 0.27 pC, *n* = 24, *p* < 0.0001, two-tailed Wilcoxon test), but significantly larger than in AP(−) (0.0136 ± 0.077 pC, *n* = 24, *p* = 0.0003, two-tailed Wilcoxon test; Figures 5C, D), indicating that release sites other than those synapsing on the recorded granule cell participate for a third of the slow current. In agreement with this spillover activation, synaptic release of GABA from unconnected pairs (for fast IPSCs) also produced small but significant Q_slow_ [AP(+): 0.11 ± 0.13 pC; AP(−):−0.039 ± 0.09 pC, *n* = 18, *p* = 0.004, two-tailed Wilcoxon test Figures 5C, D]. Overall, these data confirm that extrasynaptic high affinity GABA_A_R expressed by GrCs can be activated by spillover of GABA released from a single GoC after a single AP, even in the absence of a direct synaptic connection. Given the transmitter pooling properties of cerebellar glomeruli (Rossi and Hamann, 1998; DiGregorio et al., 2002; Crowley et al., 2009; Brandalise et al., 2012), we reasoned that the slow component could be a good indicator of the vesicular release of GABA at stimulated GoC varicosities in the glomerulus. Remarkably, GlyT2(−) GoCs carry on average 1.8 times more slow charge than GlyT2(+) pairs (0.88 ± 0.45 pC, *n* = 10, and 0.49 ± 0.33 pC, *n* = 25, respectively, *p* = 0.0298; Figure 5E).

## Discussion

### Regulation of the charge content of IPSCs at GlyT2(+) GoCs synapses by presynaptic competition of glycine with GABA

In this paper we show that the expression of the membrane transporter GlyT2 by 80% of GoCs, correlates with a reduction of the strength of their purely GABAergic transmission to GrCs. This most likely arises through presynaptic competition of glycine, accumulated by the GlyT2 transporter, with GABA for vesicular filling.

In a first step we show that increasing glycine supply decreases the charge of GrC IPSCs evoked by electrical stimulation of the GoC population. The GrC IPSC charge reduction by glycine application is characteristically linked to a reduction of the slow decay component of the phasic IPSCs. This effect is antagonized by increasing GABA supply, pointing to competition between glycine and GABA as the source of the negative impact of glycine on transmission. VIAAT is the most likely molecular site, as competition between GABA and glycine for vesicular loading by this common vesicular transporter has been demonstrated (Aubrey et al., 2007; Apostolides and Trussell, 2013). The level of vesicular loading by GABA and glycine has already been shown to control the level of postsynaptic receptor activation in the context of mixed inhibitory transmission, when probed in cultured neurons or reconstituted systems (Aubrey et al., 2007; Ishibashi et al., 2013). In these systems, high levels of cytoplasmic glycine accumulation are required to compete with GABA for vesicular loading, probably to compensate for the higher affinity of the vesicular transporter VIAAT for GABA (McIntire et al., 1997; Aubrey et al., 2007; Farsi et al., 2016). This competition for VIAAT uptake has also been quantitatively assessed by directly manipulating the cytoplasmic neurotransmitter content in acute slices and measuring the amplitude of mixed IPSCs (Apostolides and Trussell, 2013).

In a second step we perform optogenetic pairs between optogenetically stimulated GlyT2(+) or GlyT2(−) GoCs and whole-cell recorded GrCs. We find that transmission strength is scaled down in GlyT2(+) GoCs, compared to GlyT2(−) GoCs. Again, as during glycine level manipulation, this decreased strength is accompanied by a reduction of the second decay component of the fast IPSCs in GlyT2(+) GoCs. This reduction is also present in the charge of delayed IPSCs, a type of synaptic events that we identify here for the first time at this synapse, but which may relate to perisynaptic release (Huang and Bordey, 2004; Beierlein and Regehr, 2006) or to slow fusion events (Schwartz et al., 2007; Gerachshenko et al., 2009).

Interestingly, the weighted decay time constant at GlyT2(+) synapses was much more variable than at GlyT2(−) synapses in contrast to the larger variability of the amplitude and charge content at GlyT2(−) synapses. This suggests that the level of GlyT2 expression and of glycine competition with GABAergic transmission may implement a graded level of synaptic strength control within the GlyT2(+) group of GoCs. Finally, the spillover component of transmission, depending on another type of high-affinity extrasynaptic receptors, was also smaller at GlyT2(+) than at GlyT2(−) synapses, confirming a lower quantity of GABA release by GlyT2(+) GOCs.

Here, we propose that, in addition to the amplitude, the charge-to-amplitude ratio of purely GABA_A_R-mediated GrCs IPSCs is a marker of the concentration of vesicular GABA released by the presynaptic element. Kinetic modulation of IPSCs by cotransmission has previously been observed at MNTB synapses, for example, where GABA acts as a low-affinity agonist on glycine receptors and accelerates the decay of glycinergic IPSCs (Lu et al., 2008). This effect depends entirely on the activation properties of the postsynaptic receptors. Our results argue for a different mode of action, where a silent neurotransmitter (glycine) acts negatively on transmission through competition for vesicular filling with a postsynaptically active transmitter (GABA).

### GlyT2(−) GoCs as a separate functional subtype mediating granular layer spatiotemporal patterning

Converging evidence in our data points to competition of glycine with GABA for vesicular filling as the primary mechanism decreasing GABAergic transmission strength at GlyT2(+) GoCs synapses. However, the higher amplitude of GlyT2(−) IPSCs could be also partly explained, in addition to the increased vesicular GABA content, by differences in other quantal parameters like the number of contact sites or the release probability of synaptic vesicles. We find that GlyT2(−) GoCs appear to make either small connections with a high failure rate or very large connections with negligible failure. This specific patterns of strong synapses formed by each GlyT2(−) GoCs on a subset of GrCs would be ideally suited to create spatial and temporal inhibitory patterning in the granular layer (Mitchell and Silver, 2003; D’Angelo et al., 2013; Duguid et al., 2015). It is indeed likely that GlyT2(−) GoCs constitute a functional subtype, distinct from the rest of the GoC population, as previous findings indicate that GlyT2(−) GoCs are specifically contacted by mixed inhibitory neurons from the cerebellar nuclei, which can control their firing (Ankri et al., 2015). Overall, our results show that GoCs with different neurochemical profiles exert different synaptic control over the GrC population.

### Co-regulation of molecular and neurotransmitter phenotypes

The molecular diversity of GoCs extends beyond their neurotransmitter content, with the expression of various markers that appear to be co-regulated with the neurotransmitter phenotype. For instance, mGluR2 is expressed exclusively by GlyT2(+) GoCs (Simat et al., 2007), while mGluR1/5 is expressed by a small population of GoCs that is similar in abundance to GlyT2(−) cells and does not overlap with mGluR2 expression (Neki et al., 1996; Negyessy et al., 1997). Serotonin receptors are also expressed by a subpopulation of GoCs and are able to increase the level of activity in the GoC network (Fleming and Hull, 2019). Current single-cell transcriptomic data have revealed a high degree of molecular diversity within cell types (Scala et al., 2020), including cerebellar GoCs (Kozareva et al., 2021) that could be used to identify co-regulated molecular modules and highlight physiological properties underlying internal cell class diversity (Nusbaum et al., 2017), such as the putative mGluR1/5-mGluR2-GlyT2-GAD module in GoCs. However, the presents results argue for the need for detailed physiological studies to test whether molecular diversity can be interpreted as a continuum of cell properties within a class or as the substrate to define cell subclasses (Petilla Interneuron Nomenclature et al., 2008; Zeisel et al., 2015; Tasic et al., 2016; Tremblay et al., 2016; Zeng and Sanes, 2017; Saunders et al., 2018; Yuste et al., 2020).

### A push-pull hypothesis of gain control by mixed GABA/glycine inhibitory networks

The metabotropic mGluR2 operates a major inhibitory control over GoCs through massive activation of GIRK potassium channels (Watanabe and Nakanishi, 2003). GoCs mGluR2 are recruited in a graded manner by inputs from mossy fiber and parallel fiber (Watanabe and Nakanishi, 2003; Nietz et al., 2017). The mGluR2 receptors can also be activated by neighboring climbing fibers through glutamate spillover, with GoCs displaying varying degrees of inhibition (Nietz et al., 2017). Therefore, *in vivo*, sensory stimuli can cause long pauses in spontaneous firing of many GoCs (Holtzman et al., 2006b,^2011^), in part due to activation of mGluR2 (Holtzman et al., 2011). Overall, mGluR2 receptors act as a global sensor of excitatory cerebellar cortex activity in GlyT2(+) GoCs, which might be opposed by mGluR1/5 in GlyT2(−) GoCs expressing these receptors. It is therefore reasonable to propose that GoCs expressing high levels of mGluR2 could be arrested, rather than recruited, by an increased level of excitatory activity.

Based on our results on the variable synaptic strength of GoC connections, a preferential recruitment of glycine-rich cells at low levels of cerebellar activity and of GABA-rich cells at higher levels of activity would supra-linearly tune the level of inhibitory control over granule cells as a function of the overall level of MF and GrC activity, as GABA-rich cells produce large spillover and buildup components which can add up (Rossi and Hamann, 1998; Crowley et al., 2009). This organization is well suited to control the input-output relationship of the granular layer over a wide range of MF input activity (Mitchell and Silver, 2003).

Glycine may also play an opposing role in this gain control scheme, as a co-agonist of NMDA receptors (Johnson and Ascher, 1987). Potentiation of NMDA receptors by synaptically released glycine has been demonstrated in the spinal cord (Ahmadi et al., 2003). Given the low levels of D-serine in the adult cerebellar cortex (Wolosker et al., 1999; Wang and Zhu, 2003; Koga et al., 2017) and the tight control of glycine extracellular levels by glycine transporters (Supplisson and Bergman, 1997), as both GlyT2 and GlyT1 are present around and inside the cerebellar glomeruli (Zafra et al., 1995a,b), glycine released at GoCs synapses is likely the source of co-agonist for GrCs NMDA receptors. GrCs specifically express NR2C-containing NMDA receptors (Akazawa et al., 1994; Farrant et al., 1994; Monyer et al., 1994; Cathala et al., 2000) that are involved in the integration of MF input over long time scales (Schwartz et al., 2012; Powell et al., 2015; Baade et al., 2016). This integration is greatest at low MF firing rates but can saturate at high MF firing rates. A decrease in glycine-rich GoC activity during high MF activity could decrease extracellular glycine and reduce NMDA excitation of the GrC, thereby increasing the integration bandwidth of the granular layer. This NMDA/GABA push-pull action, combined with the neurochemical diversity of inhibitory populations, could be a fundamental mechanism in the lower brain to adapt, where needed, local circuit gain to the level of input activity.

## Materials and methods

### Animals

The experiments were performed on GlyT2-eGFP (Zeilhofer et al., 2005) and GlyT2-Cre transgenic mice (kind gift of HU Zeilhofer, University of Zurich) of both sexes. For the optogenetic paired recordings, GlyT2-Cre and GlyT2-eGFP mice of 6–8 weeks have been used. For the pharmacological experiments, GlyT2-eGFP mice of 5–8 weeks have been used. Mice are derived and maintained on a C57BL6/j genetic background in our animal facility. All animal manipulations were made in accordance with guidelines of the Centre national de la recherche scientifique and Use Committee.

### Stereotaxic injection

For GoC-GrC optogenetic paired recordings, cerebellar lobule IV/V of 4–5 weeks old GlyT2-Cre and GlyT2-eGFP mice were, respectively, injected with the adeno-associated viruses Mix 1 or 2, to infect GlyT2(+) GoCs or GlyT2(+) and GlyT2(−) GoCs. Mix 1: AAV2.1_CAGGS_Flex_ChR2_td-Tomato_WPRE_SV40 (titration of 1.3–5.9*10^12, Upenn Vector Core, AV-1-18917), Mix 2: AAV2.1_HSyn-Cre_WPRE_hGH (titration of 1.9–3.15*10^11, Upenn Vector Core, AV-1-PV2676) + AAV2.1_CAGGS_Flex_ChR2_td-Tomato_WPRE_SV40 (titration of 5.9*10^11, Upenn Vector Core, AV-1-18917). Mice are injected with Buprenorphine at 0.1 mg/Kg, 20–30 min before the start of the procedure. The animals are then induced with an Isoflurane/O2 mixture for 4 min at 3% and kept under anesthesia for the duration of the procedure around 1.5–2%. The correct placement of the head in the stereotaxic frame is confirmed by measuring a bregma-lambda Z deviation between 0.01 and −0.01 mm. A wide trepanation is then performed at −5.4 mm from the bregma, which reveals the bone thickening separating the colliculus from the cerebellum and the difference in brain contrast marking the transition between lobules III and IV. These internal parameters allow, if necessary, to adjust the anterior-posterior coordinates of the injection site which are likely to vary from one individual to another at this age. Injections were performed with borosilicate capillary (length: 75 mm, external diameter: 1.5 mm, thickness: 0.225 mm, Hilgenberg) filled with Mix 1 or the Mix 2. GlyT2-Cre mice received one injection (anterior-posterior: −6 mm, medio-lateral: 0 mm, dorso-ventral: −0.300 mm) and GlyT2-eGFP received two medio-lateral injection to optimize Mix 2 virus expression (anterior-posterior: −6 mm, medio-lateral: 0 ± 0.250 mm, dorso-ventral: −0.300 mm). Once in the tissue, the capillary is held for 2–3 min, then, 500 nl of virus per injection site are inoculated at constant speed (100 nl/min) and constant pressure using a Hamilton mounted on an injector. At the end of the injection, the capillary is maintained for 10 min to let the liquid diffuse into the nervous parenchyma and then removed carefully. The transgenes were let to be expressed for 2 weeks before the experiment.

### Cerebellar slices preparation

All electrophysiological experiments were performed on 300 μm tick parasagittal slices of adult mice cerebellum following the same preparation procedure. After deep anesthesia induced with isoflurane (4% isoflurane in 100% oxygen in an induction box), mice were decapitated and the cerebellum was rapidly removed and dissected in a 4°C artificial cerebrospinal fluid (ACSF) containing the following (in mM): 125 NaCl, 3.5 KCl, 1.25 NaH2PO4, 26 NaHCO3, 25 D-glucose, 1.6 CaCl2, and 1.5 MgCl2 (oxygenation 95% O2, 5% CO2). Slices are then cut using a vibrating blade microtome (Campden instrument or Leica VT 1000-S) in a potassium-rich solution (GCS) at 4°C containing the following (in mM): 130 K-gluconate, 15 KCl, 0.05 EGTA, 20 HEPES and 25 D-glucose, the pH being adjusted to 7.4 by NaOH. The slices are then transiently immersed in a modified mannitol-based recovery solution (MRS) containing (in mM): 225 D-mannitol, 2.5 KCl, 1.25 NaH2PO4, 25 NaHCO3, 25 D-glucose, 0.8 CaCl2, and 8 MgCl2 (34°C, oxygenation 95% O2, 5% CO2) to help the gradual rebalancing of the ions toward normal external concentrations. 2-amino-5-phosphonovaleric acid (D-APV, Hellobio and Tocris) at 50 μM was added to GCS and MRS to prevent glutamate excitotoxicity. Finally, the slices were transferred to oxygenated ACSF (34°C, oxygenation 95% O2, 5% CO2) in which they were stored for a maximum of 6 h. All components of ACSF, GCS, and MCS were purchased from Sigma Aldrich and the CaCl2 and MgCl2 from Fluka.

### Electrophysiology

#### Recordings

Prior to their electrophysiological recording, slices are transferred to a recording chamber mounted on an upright microscope (Olympus) and perfused (4 ml/min) with oxygenated (95% O2, 5% CO2) at a temperature of 32–34°C under the objective. The slices were visualized thanks to an infrared-light source, a 20× immersion objective (XLUM Plan FI, Olympus) and a camera (Cool Snap HQ, Photometrics). The recording and stimulation pipettes were stretched from borosilicate glass capillaries (length: 75 mm, outer diameter: 1.5 mm, wall thickness: 0.225 mm, Hilgenberg) with a home-made vertical puller. GoCs are identified in slices by the expression of GFP in GlyT2-eGFP and/or Tomato in injected mice. GrCs are easily recognized by their size, fast mono-exponential capacitive current and a capacitance <4 pF (Silver et al., 1992). The effect of optogenetic stimulations on targeted GoCs are recorded in loose-cell-attached (holding at 0 mV) with 3–4 MΩ electrodes filled with an intrapipette solution containing (in mM): 140 NaCl, 2.34 KCl, 1.25 NaH2PO4, 10 HEPES, 1.3 CaCl2, 1.1 MgCl2, pH adjusted to 7.4 with 1 M NaOH. For all experiments, GrCs are recorded in whole cell configuration and voltage clamp mode (holding at −70 mV) with 7–8 MΩ electrodes filled with an intracellular solution containing (in mM): 110 CsCl, 20 TEA-Cl, 10 HEPES, 6 NaCl, 10 EGTA, 0.2 CaCl2, 4 ATP-Mg, 0.4 GTP-Na, pH adjusted to 7.4 with CsOH at 1 M. The data were acquired with an EPC10 amplifier (HEKA), sampled at 20 KHz and filtered at 8 or 3 KHz.

#### Optogenetic stimulation of GoCs

The source of light for optogenetic stimulations was a 470 nm LED (M470F3, THORLABS) relayed to the sample by a collimation lens system and a lateral port of the Olympus equipped with the proper dichroic mirror. This optical setup allows to create a near-collimated spot of 20–30 μm in diameter on the sample. The optogenetic stimulation was delivered in 2 ms flashes which intensity was controlled linearly by the amplitude of the analog voltage step generated by the EPC10 interface (HEKA). The threshold for optogenetic stimulation intensity to GoCs was explored manually using a LEDD1B controller (THORLABS). The frequency of the optogenetic stimulation for synaptic transmission characterization in pairs was 0.37 Hz.

#### Electrical stimulation of the GoCs axons

A current generator (Isostim™ A320, WPI) driven by the EPC10 amplifier has been used to deliver minimal electrical stimulations of 0.3 ms to the GoCs axons in GlyT2-eGFP slices. The stimulation electrode (7–8 MΩ) was filled with the same solution as for the loose-cell-attached recordings. The stimulation electrodes were systematically placed 50–100 μm away from the recorded GrC to avoid direct stimulation. The stimulation frequency was set at 10 Hz in continue for vesicular content manipulation experiments.

#### Pharmacology

For the recording of the GoC-GrC optogenetic pairs, D-APV 50 μM, NBQX 2 μM (Hellobio and Tocris) and strychnine 0.5 μM (Sigma Aldrich) was added to the ACSF to avoid uncontrolled activation and inhibition of GoCs and isolate pure GABAergic IPSCs in our high chloride recording condition. The GABAergic IPSCs evoked in GrCs by the repetitive electrical stimulation of the GoCs axons have been isolated by adding D-APV 50 μM, NBQX 2 μM, strychnine 0.5 μM to avoid activation of glycine receptors expressed by GoCs (Dumoulin et al., 2001) and CGP55845 1 μM (Abcam) to avoid GABA_B_R activation in this condition (Mapelli et al., 2009). In some experiments, the ACSF was supplemented with 500 μM of glutamine (Sigma Aldrich) to increase *de novo* synthesis of GABA (Wang et al., 2013). Continuous repetitive 10 Hz stimulations of GoCs in adult slices led to rundown of the GABAergic transmission after 10 min of recording in classical slices preparation (39 ± 24% of loss; *n* = 12). To stabilize the synaptic transmission during 10 Hz train stimulation, we improved the supply of essential substrates for general neuronal metabolism by incubating slices for at least 1 h in BrainPhys (StemCell), a specially adapted supplemented culture medium (Bardy et al., 2015). In these conditions, the rundown was reduced to 20 ± 27% (no BrainPhys incubation (*n* = 12) vs. BrainPhys incubation (*n* = 22): *p* = 0.015, Supplementary Figure 1B). Due to the remaining rundown of the transmission over the time and its cell-to-cell variability, we randomly apply glycine (100 μM, 5 min, Sigma Aldrich) after 3 min of baseline recording. To avoid the buffering of the applied glycine by glial cells and that they became an uncontrolled source of glycine during washout, the glial transporter of glycine (GlyT1b) was blocked continuously with of 1 μM of ORG24598 (Tocris) in all pharmacological experiments (Brown et al., 2001). In experiments of Figure 1H, the neuronal transporter of glycine (GlyT2) has been blocked by adding 1 μM of ORG25543 (Tocris) (Caulfield et al., 2001) during the recovery and the experiment. All drugs were applied in the recording chamber *via* the infusion system at the same rate and temperature as ASCF (4 ml/min, 32–34°C).

#### Image acquisition

The images of the infected and recorded slices were all acquired with an inverted confocal microscope equipped with a white laser (SP8, Leica).

### Analysis

#### Events detection and classification

The recordings were analyzed with algorithms developed on Python (Python Software Foundation, version 2.7). The method to differentiate connected from not connected pairs and transmission successes from failures are detailed in the text. Transmission successes are further segregated in phasic and delayed IPSCs based on the lack of the fast-rising and fast-decaying component in the latter. The difference in the rising phase kinetic has been measured with a sliding difference between the mean of a 10 ms time window and the mean of a 0.5 ms one, both separated by 0.3 ms corresponding to the rising time of a classical fast IPSC. The time to the maximal difference for a pair was set as the fast component rising time. Then, a 0.5 ms jitter of GoC-GrC transmission delay (Dugue et al., 2005) was added around the fast component rising time previously measured to create, for each pair, the time window in which all fast-rising IPSCs should fall. An IPSC success was classed as phasic when the maximal difference in that window was superior to the baseline +2SD, otherwise it was classed as delayed IPSCs. The kinetics analysis of the IPSCs decay time were performed on Clampfit (Molecular Devices, San Jose, CA, United States) from averaged traces.

#### Statistics

The statistics were made with the SciPy library available on Python (Virtanen et al., 2020). The data are presented as mean ± SD unless otherwise stated in the text. All population difference significance was assessed by a bilateral non-parametric Mann-Whitney ranked test due to the small size of our groups and their no monotone distribution. Paired comparisons were calculated by a non-parametric Wilcoxon bilateral test when appropriated. All correlations have been calculated with the Spearman method and the corresponding two-sided *p*-value were calculated with a t statistic. When correlations are significant, they are represented with the linear regression on the figure and the slope is reported in the text. The significance of the variance difference between GlyT2(+) and GlyT2(−) pairs have been tested by running a Conover test of equal variance (Wolfram function on Mathematica 12). An effect is considered significant if the *p*-value is less than 0.05. A *p*-value is considered as strong when below 0.0001 and is reported as <0.0001 in the text for clarity.

#### Sample size estimation

In accordance with previous studies using cerebellar and brain stem slices (Stell et al., 2003; DiGregorio et al., 2007; Crowley et al., 2009; Bright et al., 2011; Hirono et al., 2012; Apostolides and Trussell, 2013; Ankri et al., 2015; Szoboszlay et al., 2016; Fleming and Hull, 2019; Schonewille et al., 2021) and the central limit theorem from the high number theory, we tried to reach a number of independent biological replicates (all “n” in this study) around 10 to 30 in each experiments.

#### Attrition

Instable recordings from GrCs and spontaneous activity in GoCs were the only exclusion parameters used in this study. For the analysis of the optogenetic pairs, group size change along the study because the amount of recording in different class of event varies between pairs. Seven pairs [2 GlyT2(−) and 5 GlyT2(+)] did not have enough delayed IPSCs to be properly fitted with a mono-exponential function. Two GlyT2(+) pairs had a too small signal-to-noise ratio to be properly fitted with the two exponential function and could not be used for the quantification of Q_AP(+)_ and Qslow_AP(+)_. Another GlyT2(+) pair shows a marked artifactual rupture in its AP(+) current integral after 100 ms and were thus removed from Qslow_AP(+)_ analysis.

## Data availability statement

The datasets presented in this study can be found online in the dryad online repository (https://datadryad.org) with the doi: 10.5061/dryad.2rbnzs7qs.

## Ethics statement

The animal study was reviewed and approved by the French National Ethic Committee for Sciences and Health report on “Ethical Principles for Animal Experimentation” in agreement with the European Community Directive 86/609/EEC under agreement #12007, from the “Darwin” committee.

## Author contributions

DD contributed to the conceptualization, investigation, methodology, data curation, formal analysis, software, visualization, writing original draft, and writing review and editing. CM-H contributed to the investigation and methodology. SS contributed to the formal analysis, supervision, writing original draft, and writing review and editing. SD contributed to the conceptualization, data curation, formal analysis, methodology, resources, supervision, validation, funding acquisition, project administration, writing original draft, and writing review and editing. All authors contributed to the article and approved the submitted version.
