# Supplementary material for: Neurotransmitter content heterogeneity within an interneuron class shapes inhibitory transmission at a central synapse
Source: Front Cell Neurosci. 2023 Jan 4;16:1060189. doi: 10.3389/fncel.2022.1060189 (PMC9846633; doi:10.3389/fncel.2022.1060189)
Supplement: Supplementary file 1 [file Data_Sheet_1.PDF]

*Supplementary Material*

# Neurotransmitter content heterogeneity within an interneuron class shapes inhibitory transmission at a central synapse

**Dimitri Dumontier[1]†, Caroline Mailhes-Hamon[1]‡, Stéphane Supplisson[1]§, Stéphane Dieudonné[1]¶***

1 : Institut de Biologie de l'Ecole Normale Supérieure (IBENS), École normale supérieure, Université PSL, CNRS, Inserm, Paris, France

ORCID : † 0000-0002-5515-2162; ‡0000-0002-7009-7798; §0000-0002-0062-9752 ; ¶0000-0001-5336-8894

*Correspondence : dieudon@bio.ens.psl.eu

**Supplementary Figure 1**. A, Cartoon illustrating the working hypothesis for our pharmacological experiment around Glycine application. B, Effect of BrainPhys pre-incubation of adult cerebellar slices on Golgi cells GABAergic transmission during the continuous electrical stimulation of their axons at 10 Hz. Top panel, two individual examples showing 100 averaged GABAergic eIPSCs every 20 seconds for 10 minutes when incubated in BrainPhys (black) or in standard ACSF (red). This spaced representation of averaged eIPSCs aims at showing more clearly the kinetics of the events over time. Bottom panel, quantification of the GABAergic transmission strength over the time with or without slices incubation in BrainPhys. The strength of the GABAergic transmission is estimated by taking the charge (QeIPSC) of the averaged eIPSCs (shown on the top panel) every 10 seconds. This charge is then normalized by the baseline charge (3 minutes from the beginning). The two curves are the populational GABAergic transmission over time after incubation in BrainPhys (black, n = 22) or in ASCF (red, n = 12) ± SEM. C, left panel, Populational averaged T1 and T2 eIPSCs in ACSF with or without Glycine application. Right panel, Corresponding peak normalized traces. D, same as C in

ACSF + ORG25543, the specific blocker of GlyT2. E, same as C and D in ACSF supplemented with 500 µM of Glutamine (the precursor of the GABA synthesis).
