# Supplementary figures and images for: Neurotransmitter content heterogeneity within an interneuron class shapes inhibitory transmission at a central synapse
Source: Front Cell Neurosci. 2023 Jan 4;16:1060189. doi: 10.3389/fncel.2022.1060189 (PMC9846633; doi:10.3389/fncel.2022.1060189)

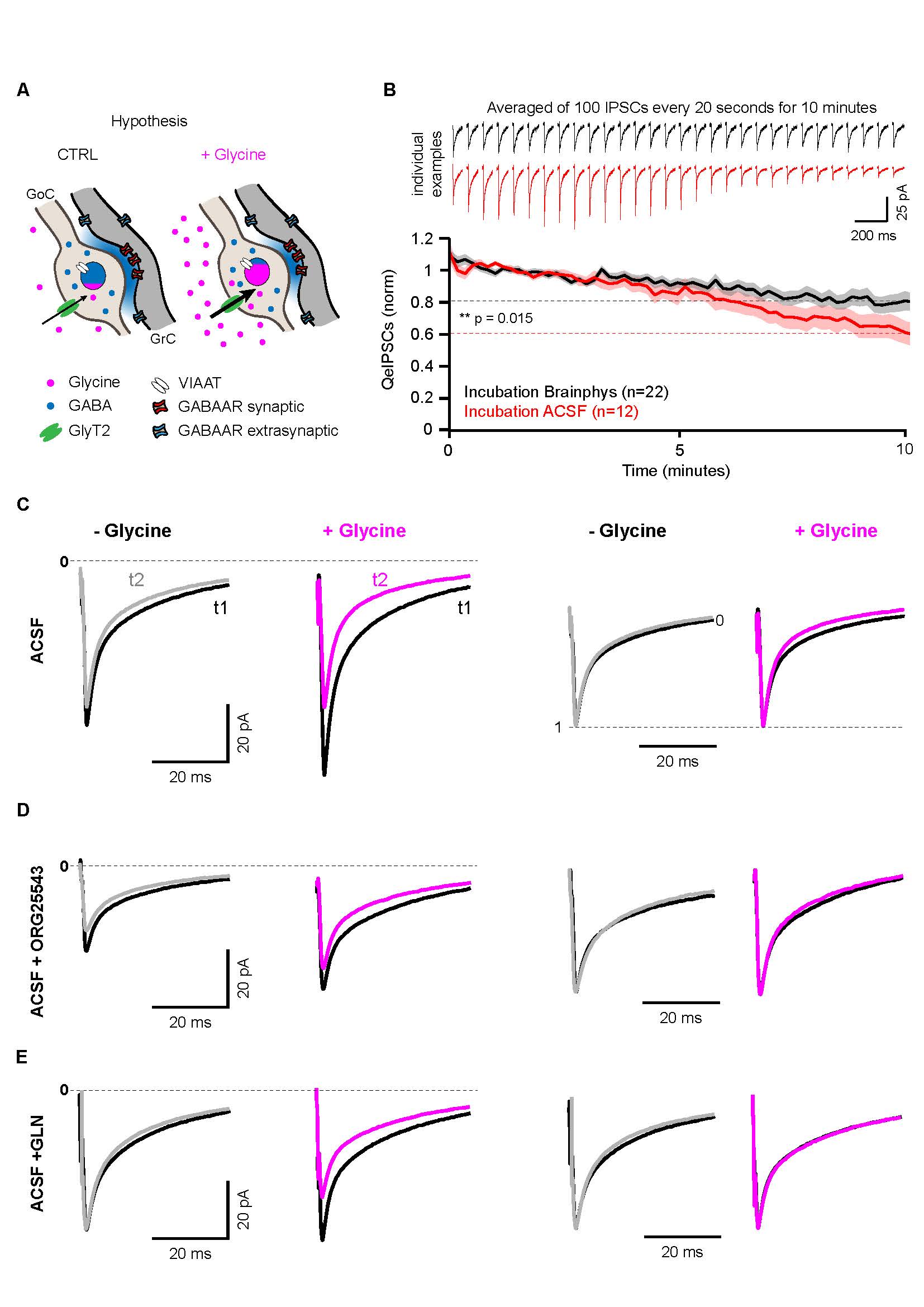

Supplement: Supplementary file 2 [file Image_1.JPEG]
